# Operative care rates of lateral compression type 1 pelvic fractures increased from 2003 to 2018

**DOI:** 10.1007/s00590-026-04900-0

**Published:** 2026-08-01

**Authors:** Daniel Rusu, Andrew Duong, Sagar Telang, Nathanael Heckmann, Joseph Patterson

**Affiliations:** 1 Department of Orthopaedic Surgery, Keck School Medicine of USC, Los Angeles, USA; 2 Department of Orthopaedic Surgery, University of Arizona College of Medicine- Phoenix, Phoenix, USA; 3 Department of Orthopaedic Surgery, University of California, Davis, Sacramento, USA

**Keywords:** Pelvis fracture, LC1, Operative management, Mortality, Practice, Temporal trend

## Abstract

**Purpose:**

To investigate trends in operative management of LC1 pelvic fractures and potential effects on hospital length of stay, home discharge, and mortality.

**Methods:**

The National Inpatient Sample (NIS) was queried and identified 43,743 older adults aged ≥ 60 years and 29,024 younger adults aged 18–59 years who had LC1 fractures via ICD diagnosis codes (2003–2018). Operative management was classified by ICD procedure codes for internal and/or external fixation. Adjusted annual change in rate of operative care, length of stay, mortality, and home discharge were assessed using multivariable robust Poisson regression models.

**Results:**

Operative care was provided to 1413 (3.2%) older adults and 5871 (20.2%) younger adults. Among older adults, operative care increased 5% per year (RR per year: 1.05, 95% CI 1.03–1.06, *p* < 0.001), with relative per year decreases in length of stay (RR per year: 0.98, 95% CI 0.98–0.98, *p* < 0.001), home discharge (RR per year: 0.99, 95% CI 0.99–1.00, *p* = 0.02) and mortality (RR per year: 0.95, 95% CI 0.95–0.96, *p* < 0.001). Among younger adults, operative care increased 2% per year (RR per year: 1.02, 95% CI 1.01–1.02, *p* < 0.001), with relative per year decreases in length of stay (RR per year: 0.98, 95% CI 0.98–0.98, *p* < 0.001), and mortality (RR per year: 0.95, 95% CI 0.94–0.96, *p* < 0.001).

**Conclusion:**

Operative care of LC1 fractures in the United States increased from 2003 to 2018. A rise in operative care was associated with decreases in length of stay and mortality in older and younger adults, but fewer home discharges for older adults.

**Level of evidence:**

Level III Therapeutic.

**Supplementary Information:**

The online version contains supplementary material available at 10.1007/s00590-026-04900-0.

## Introduction

Lateral compression type 1 (LC1) pelvic fractures, AO/OTA 61B2.1, constitute nearly 2/3 of all pelvic ring injuries and affect 600,000 persons each year [1]. These injuries may be caused by high-energy trauma, typically affecting younger patients, or low-energy ground level falls, typically affecting older individuals [1]. With an aging United States (US) population, the incidence of LC1 fractures amongst older adults is increasing and expected to be a growing mortality and morbidity [2]. The mortality rate of LC1 pelvic fractures during the index hospital admission is reported to be between 5% and 9%, while the one-year mortality rate is 21% [3, 4]. Survivors of LC1 fractures report decreased independence in daily living, emotional well-being, and quality of life [5].

LC1 fractures have been considered stable injuries and historically managed nonoperatively [6, 7]. Nonoperative management typically results in satisfactory radiographic functional outcomes after nonoperative management [8, 9]. However, LC1 fractures encompass a wide range of injuries from stable, nondisplaced sacral fractures to displaced, unstable sacral fractures with associated anterior pelvic ring injuries [1]. Recent investigations suggest that operative internal fixation may improve patient outcomes and limit healthcare costs by decreasing pain with mobilization, improving ambulation, shortening hospital stay, decreasing late operations for fracture-related complications, increasing healthy days at home after treatment, and reducing mortality [9–13]. It is not known if these observations have influenced practice or resulted in meaningful changes in patient outcomes at a national level.

The purpose of this investigation was to describe a recent trend in operative versus nonoperative care and associated outcomes of LC1 fractures. The hypothesis was that operative care of LC1 fractures increased year-to-year, which may reflect a response to emerging evidence advocating for operative care of these injuries. Secondary objectives included determining if operative care was associated with patient-identified outcome priorities of in-hospital mortality, length of hospital stay, or discharge to home [14].

## Methods

### Patient selection

A retrospective database cohort study was undertaken using the publicly available NIS curated by the Healthcare Cost and Utilization Project. NIS data describe demographics, diagnoses, procedures, outcomes, charges, and healthcare utilization for over 7 to 8 million hospital discharges per year, representing approximately 20% of all discharges across both community and academic hospitals in the United States. Adult patients (age ≥ 18 years) admitted between January 1st, 2003 and December 31st, 2018 with diagnoses consistent with a closed LC1 pelvic fracture pattern were identified using International Classification of Diseases Ninth Edition (ICD-9) and Tenth Edition (ICD-10) Clinical Modification diagnosis codes (Supplementary Table 1). Operative care was identified using ICD-9 and ICD-10 procedure codes for surgical treatment of pelvic fractures (Supplementary Table 2). The diagnosis and procedure code logic used to select a sample of LC1 fractures were iteratively tested on institutional data with radiographic confirmation of the correct procedure and fracture pattern before finalizing the NIS query.

### Outcomes

The primary outcome was the adjusted relative annual change in the portion of LC1 fractures treated with operative versus nonoperative care. Secondary outcomes included the adjusted relative annual change in rate of mortality, length of stay, and rate of home discharge.

### Statistical methods

Patients were stratified by age 18–59 (younger adults) or ≥ 60 (older adults). Linear regression models were used to assess the relative annual change in unadjusted rates of operative treatment, mortality, home discharge, and the change in length of stay (days). To address potential confounding by putatively associated variables with operative care, multivariable models were constructed including demographics, pre-existing comorbidities, primary payor, treating hospital characteristics, and All Patients Refined Diagnosis Related Groups (APR-DRG) Risk of Mortality and Severity of Illness scores. Robust Poisson regression models were applied with calendar year as a categorical variable to determine adjusted rates of operative treatment, home discharge, and mortality with 2003 serving as the reference year and using year of treatment as a continuous variable to derive an adjusted annual change in rate. As overdispersion was found in length of stay analysis, a multivariable negative binomial regression model used to assess temporal trends in hospital stay. Sequential minimization of the Akaike Information Criterion (AIC) was used to optimize covariate selection. A *p*-value of < 0.05 was considered significant. APR-DRG data for 2015 was missing and was thus imputed by ordinal logistic regression imputation using variables included after AIC minimization as input. Subsequently, any patient with missing data was excluded from analysis. Statistical tests were performed using R statistical software (R Foundation, Vienna, Austria).

## Results

A total of 72,767 patients met inclusion criteria consistent with an LC1 fracture pattern. Of these, 43,743 (60.1%) were ≥ 60 years old and 29,024 (39.9%) were 18–59 years old.

### Younger adults

Younger adults received operative care in 20.2% of admissions. Young adults treated operatively were younger with a mean age of 36.6 vs. 37.7 years (*p* < 0.001), more commonly male (62.5% vs. 48.8%, *p* < 0.001), less likely to have 12 of the 17 comorbid medical conditions evaluated (absolute percent difference range = 0.1–1.9%), but significantly more likely to have peptic ulcer disease and peripheral vascular disease (absolute percent difference range = 0.1–0.3%). Younger adults treated operatively were more likely to have private insurance (51.2% vs. 50.4%, *p* < 0.001). They were more likely to receive care at larger hospitals (79.5% vs. 71.0%, *p* < 0.001). Operative care was more likely provided at urban teaching hospitals (87.7% vs. 73.5%, *p* < 0.001). Geographically, operative treatment was more commonly administered in the Southern United States (48.1% vs. 44.2% *p* < 0.001). Per the APR-DRG classification, younger adults treated operatively were more frequently classified as having a major or extreme severity of illness (76.7% vs. 56.9%, *p* < 0.001) and moderate or greater risk of mortality (62.4% vs. 48.9%, *p* < 0.001; Table 1).

**Table 1:** Demographic, Comorbidity, Payer, and Diagnosis Characteristics

Patients aged ≥ 60 years	Full cohort *N* = 43,743 (%)	Non-operative *N* = 42,290 (%)	Operative *N* = 1,453 (%)	*P *value
Demographics				
Mean age, years	79.3 (95% CI 61.0–97.7)	79.6 (95% CI 61.5–97.8)	70.4 (95% CI 53.7–87.0)	< 0.001
Sex				
Male	9,130 (20.9%)	8292 (19.6%)	838 (57.7%)	< 0.001
Female	34,613 (79.1%)	33,998 (80.4%)	615 (42.3%)	
Comorbidities				
CCI score, median	4 (IQR: 2)	5 (IQR: 2)	3 (IQR: 2)	< 0.001
AIDS/HIV	11 (< 0.1%)	10 (< 0.1%)	1 (0.1%)	0.981
Metastatic solid tumor	575 (1.3%)	572 (1.4%)	3 (0.2%)	< 0.001
Liver disease				
Mild	1111 (2.5%)	1069 (2.5%)	42 (2.9%)	0.004
Moderate/severe	447 (1.0%)	424 (1.0%)	23 (1.6%)	0.184
Malignancy	1780 (4.1%)	1759 (4.2%)	21 (1.4%)	< 0.001
Renal	6117 (14.0%)	6015 (14.2%)	102 (7.0%)	< 0.001
Hemiplegia/Paraplegia	380 (0.9%)	367 (0.9%)	13 (0.9%)	0.663
Diabetes				
With complication	3612 (8.3%)	3486 (8.2%)	126 (8.7%)	< 0.001
Without complication	7112 (16.3%)	6884 (16.3%)	228 (15.7%)	< 0.001
PUD	376 (0.9%)	370 (0.9%)	6 (0.4%)	0.003
Rheumatic	2042 (4.7%)	2011 (4.8%)	31 (2.1%)	< 0.001
COPD	9180 (21.0%)	8991 (21.3%)	189 (13.0%)	< 0.001
Dementia	4638 (10.6%)	4583 (10.8%)	55 (3.8%)	< 0.001
CVD	2446 (5.6%)	2393 (5.7%)	53 (3.6%)	< 0.001
PVD	4694 (10.7%)	4577 (10.8%)	117 (8.1%)	< 0.001
CHF	6336 (14.5%)	6244 (14.8%)	92 (6.3%)	< 0.001
MI	2634 (6.0%)	2562 (6.1%)	72 (5.0%)	< 0.001
Expected primary payer				
Medicare	35,770 (81.8%)	35,072 (82.9%)	698 (48.0%)	< 0.001
Medicaid	746 (1.7%)	676 (1.6%)	70 (4.8%)	
Private insurance	5780 (13.2%)	5247 (12.4%)	533 (36.7%)	
Self-pay	500 (1.1%)	458 (1.1%)	42 (2.9%)	
No charge	37 (0.1%)	32 (0.1%)	5 (0.3%)	
Other	910 (2.1%)	805 (1.9%)	105 (7.2%)	
Region of hospital				
Northeast	8467 (19.4%)	8269 (19.6%)	198 (13.6%)	< 0.001
Midwest	8480 (19.4%)	8271 (19.6%)	209 (14.4%)	
South	17,648 (40.3%)	16,993 (40.2%)	655 (45.1%)	
West	9148 (20.9%)	8757 (20.7%)	391 (26.9%)	
Location/teaching status of hospital				
Rural	4767 (10.9%)	4739 (11.2%)	28 (1.9%)	< 0.001
Urban nonteaching	14,333 (32.8%)	14,184 (33.5%)	149 (10.3%)	
Urban teaching	24,643 (56.3%)	23,367 (55.3%)	1276 (87.8%)	
Bedsize of hospital				
Small	6831 (15.6%)	6765 (16.0%)	66 (4.5%)	< 0.001
Medium	11,528 (26.4%)	11,271 (26.7%)	257 (17.7%)	
Large	25,384 (58.0%)	24,254 (57.4%)	1130 (77.8%)	
APR-DRG classification				
Severity of illness				
Minor	2477 (5.7%)	2440 (5.8%)	37 (2.5%)	< 0.001
Moderate	17,011 (38.9%)	16,678 (39.4%)	333 (22.9%)	
Major	19,715 (45.1%)	19,105 (45.2%)	610 (42.0%)	
Extreme	4540 (10.4%)	4067 (9.6%)	473 (32.6%)	
Risk of mortality				
Minor	9181 (21.0%)	8803 (20.8%)	378 (26.0%)	< 0.001
Moderate	21,218 (48.5%)	20,796 (49.2%)	422 (29.0%)	
Major	9912 (22.7%)	9555 (22.6%)	357 (24.6%)	
Extreme	3432 (7.8%)	3136 (7.4%)	296 (20.4%)	
Mortality	1515 (3.5%)	1460 (3.5%)	55 (3.8%)	< 0.001
Length of stay	4 (IQR: 4)	4 (IQR: 4)	9 (IQR: 10)	< 0.001

Patients aged 18–59 years	Full cohort *N* = 29,024 (%)	Non-operative *N* = 23,153 (%)	Operative *N* = 5,871 (%)	*P* value
Demographics				
Mean age, years	37.5 (95% CI 11.1–63.9)	37.7 (95% CI 11.0–64.4)	36.6 (95% CI 11.5–61.7)	< 0.001
Sex				
Male	14,970 (51.6%)	11,298 (48.8%)	3672 (62.5%)	< 0.001
Female	14,054 (48.4%)	11,855 (51.2%)	2199 (37.5%)	
Comorbidities				
CCI score, median	0 (IQR: 1)	0 (IQR: 1)	0 (IQR: 1)	< 0.001
AIDS/HIV	53 (0.2%)	47 (0.2%)	6 (0.1%)	0.981
Metastatic solid tumor	95 (0.3%)	89 (0.4%)	6 (0.1%)	< 0.001
Liver disease				
Mild	778 (2.7%)	668 (2.9%)	110 (1.9%)	0.004
Moderate/severe	302 (1.0%)	261 (1.1%)	41 (0.7%)	0.184
Malignancy	234 (0.8%)	213 (0.9%)	21 (0.4%)	< 0.001
Renal	660 (2.3%)	593 (2.6%)	67 (1.1%)	< 0.001
Hemiplegia/Paraplegia	376 (1.3%)	317 (1.4%)	59 (1.0%)	0.663
Diabetes				
With complication	722 (2.5%)	624 (2.7%)	98 (1.7%)	< 0.001
Without complication	1449 (5.0%)	1201 (5.2%)	248 (4.2%)	< 0.001
PUD	90 (0.3%)	69 (0.3%)	21 (0.4%)	0.003
Rheumatic	270 (0.9%)	243 (1.0%)	27 (0.5%)	< 0.001
COPD	2203 (7.6%)	1842 (8.0%)	361 (6.1%)	< 0.001
Dementia	121 (0.4%)	101 (0.4%)	20 (0.3%)	< 0.001
CVD	412 (1.4%)	347 (1.5%)	65 (1.1%)	< 0.001
PVD	521 (1.8%)	404 (1.7%)	117 (2.0%)	< 0.001
CHF	366 (1.3%)	327 (1.4%)	39 (0.7%)	< 0.001
MI	242 (0.8%)	207 (0.9%)	35 (0.6%)	< 0.001
Expected primary payer				
Medicare	1683 (5.8%)	1508 (6.5%)	175 (3.0%)	< 0.001
Medicaid	5305 (18.3%)	4186 (18.1%)	1119 (19.1%)	
Private insurance	14,665 (50.5%)	11,661 (50.4%)	3004 (51.2%)	
Self-pay	3798 (13.1%)	3054 (13.2%)	744 (12.7%)	
No charge	288 (1.0%)	226 (1.0%)	62 (1.1%)	
Other	3285 (11.3%)	2518 (10.9%)	767 (13.1%)	
Region of hospital				
Northeast	4407 (15.2%)	3615 (15.6%)	792 (13.5%)	< 0.001
Midwest	4669 (16.1%)	3756 (16.2%)	913 (15.6%)	
South	13,054 (45.0%)	10,228 (44.2%)	2826 (48.1%)	
West	6894 (23.8%)	5554 (24.0%)	1340 (22.8%)	
Location/teaching status of hospital				
Rural	1270 (4.4%)	1205 (5.2%)	65 (1.1%)	< 0.001
Urban nonteaching	5585 (19.2%)	4925 (21.3%)	660 (11.2%)	
Urban teaching	22,169 (76.4%)	17,023 (73.5%)	5146 (87.7%)	
Bedsize of hospital				
Small	1868 (6.4%)	1681 (7.3%)	187 (3.2%)	< 0.001
Medium	6042 (20.8%)	5025 (21.7%)	1017 (17.3%)	
Large	21,114 (72.7%)	16,447 (71.0%)	4667 (79.5%)	
APR-DRG classification				
Severity of illness				
Minor	1538 (5.3%)	1396 (6.0%)	142 (2.4%)	< 0.001
Moderate	9806 (33.8%)	8575 (37.0%)	1231 (21.0%)	
Major	11,688 (40.3%)	9148 (39.5%)	2540 (43.3%)	
Extreme	5992 (20.6%)	4034 (17.4%)	1958 (33.4%)	
Risk of mortality				
Minor	14,047 (48.4%)	11,841 (51.1%)	2206 (37.6%)	< 0.001
Moderate	7753 (26.7%)	6107 (26.4%)	1646 (28.0%)	
Major	4330 (14.9%)	3121 (13.5%)	1209 (20.6%)	
Extreme	2894 (10.0%)	2084 (9.0%)	810 (13.8%)	
Mortality	898 (3.1%)	814 (3.5%)	84 (1.4%)	< 0.001
Length of stay	6 (IQR: 8)	5 (IQR: 7)	10 (IQR: 11)	< 0.001

CCI, Charlson Comorbidity Index; PUD, Peptic Ulcer Disease; COPD, Chronic Obstructive Pulmonary Disease; CVD, Cardiovascular Disease; PVD, Peripheral Vascular Disease; CHF, Congestive Heart Failure; MI, Myocardial Infarction; APR-DRG, All Patients Refined Diagnosis Related Groups

The in-hospital mortality rate among younger adults was 3.1%. The discharge to home rate was 61.8%. The median length of stay was 6 days (IQR = 8 days). Younger adults who received operative care stayed in the hospital longer (median 10 vs. 5 days, *p* < 0.001), were less frequently discharged home (49.0% vs. 65.1%, *p* < 0.001), and experienced less mortality (1.4% vs. 3.5%, *p* < 0.001; Table 1).

The portion of younger adults treated operatively increased 0.46% per year (95% CI 0.14–0.79, *p* = 0.01). The home discharge rate decreased − 0.32% per year (95% CI − 0.58 to − 0.06, *p* = 0.01). No significant temporal trends in unadjusted length of stay or mortality were observed.

Multivariable analysis demonstrated that the adjusted annual rate of operative care increased by 1.9% per year (95% CI 1.014–1.024, *p* < 0.001) among younger adults. Adjusted length of stay and adjusted mortality decreased by 1.6% (95% CI 0.981–0.987, *p* < 0.001) and 4.7% (95% CI 0.943–0.962, *p* < 0.001), respectively. Adjusted home discharge rates did not significantly vary over the observed period (95% CI 0.998–1.001, *p* = 0.803; Table 2).

**Table 2:** Comparison of Multivariable Poisson Regression Models Assessing Patients Between 18 to 59 years

Patients aged 18–59 years	Dependent variable			
	Home discharge (1)	LOS (2)	Operative care (3)	Mortality (4)
Home discharge		0.843*** (95% CI 0.819–0.868)	0.658*** (95% CI 0.627–0.690)	
LOS	0.993*** (95% CI 0.991–0.995)		1.007*** (95% CI 1.006–1.009)	0.929*** (95% CI 0.914–0.944)
Operative care	0.824*** (95% CI 0.802–0.846)	1.224*** (95% CI 1.188–1.260)		0.478*** (95% CI 0.383–0.595)
Mortality			0.296*** (95% CI 0.239–0.367)	
Demographics				
Age	0.993*** (95% CI 0.992–0.993)	1.006*** (95% CI 1.005–1.008)		1.009** (95% CI 1.005–1.013)
Female	0.964* (95% CI 0.949–0.981)	0.928*** (95% CI 0.905–0.953)	0.730*** (95% CI 0.696–0.765)	0.825** (95% CI 0.752–0.906)
Comorbidities				
CCI Score		0.940*** (95% CI 0.911–0.970)	0.865*** (95% CI 0.835–0.896)	0.912* (95% CI 0.852–0.976)
AIDS/HIV		1.277 (95% CI 0.928–1.758)		
Metastatic solid tumor	1.262 (95% CI 1.076–1.480)	1.276*** (95% CI 0.955–1.706)		3.886* (95% CI 1.178–12.814)
Liver disease				
Mild		1.071*** (95% CI 0.945–1.213)		1.445* (95% CI 1.130–1.849)
Moderate/severe		1.144** (95% CI 0.946–1.385)		1.715** (95% CI 1.292–2.277)
Malignancy		1.086*** (95% CI 0.909–1.297)		
Renal		1.121*** (95% CI 1.007–1.249)	0.794 (95% CI 0.624–1.010)	1.399 (95% CI 0.982–1.994)
Hemiplegia/Paraplegia	0.740*** (95% CI 0.651–0.841)	1.466*** (95% CI 1.296–1.658)	0.777 (95% CI 0.604-1.000)	
Diabetes				
With complication		1.114*** (95% CI 1.008–1.232)		
Without complication			1.284** (95% CI 1.130–1.459)	
PUD		1.270* (95% CI 1.060–1.522)		
COPD	1.049 (95% CI 1.016–1.083)	0.986*** (95% CI 0.926–1.050)	1.099 (95% CI 0.991–1.218)	0.782 (95% CI 0.598–1.022)
Dementia	0.758 (95% CI 0.602–0.955)	1.501*** (95% CI 1.256–1.793)		
CVD	0.839* (95% CI 0.742–0.947)	1.178*** (95% CI 1.042–1.333)	0.804 (95% CI 0.642–1.008)	
PVD		0.955*** (95% CI 0.876–1.042)		
CHF		1.126*** (95% CI 0.988–1.284)		
MI		1.032*** (95% CI 0.883–1.206)		
Expected primary payer				
Medicaid	1.326*** (95% CI 1.255–1.401	1.326*** (95% CI 1.246–1.412)	1.512*** (95% CI 1.305–1.752)	1.471* (95% CI 1.124–1.927)
Private insurance	1.215*** (95% CI 1.152–1.281)	1.054 (95% CI 0.996–1.116)	1.657*** (95% CI 1.439–1.908)	1.137 (95% CI 0.876–1.477)
Self-pay	1.570*** (95% CI 1.488–1.657)	1.126*** (95% CI 1.054–1.202)	1.583*** (95% CI 1.358–1.846)	1.702** (95% CI 1.293–2.239)
No charge	1.449*** (95% CI 1.333–1.576)	1.259** (95% CI 1.078–1.471)	1.506** (95% CI 1.167–1.943)	1.264 (95% CI 0.620–2.576)
Other	1.220*** (95% CI 1.152–1.292)	1.094** (95% CI 1.025–1.168)	1.725*** (95% CI 1.482–2.007)	1.171 (95% CI 0.870–1.575)
Region of hospital				
Midwest	1.104*** (95% CI 1.067–1.142)	0.811*** (95% CI 0.776–0.848)	1.165** (95% CI 1.073–1.265)	1.040 (95% CI 0.868–1.247)
South	1.273*** (95% CI 1.238–1.309)	0.958*** (95% CI 0.920–0.997)	1.292*** (95% CI 1.206–1.384)	1.346** (95% CI 1.160–1.562)
West	1.163*** (95% CI 1.128–1.199)	0.880*** (95% CI 0.842–0.920)	1.206*** (95% CI 1.116–1.303)	1.133 (95% CI 0.956–1.342)
Location/teaching status of hospital				
Urban nonteaching	0.920* (95% CI 0.887–0.955)	1.035 (95% CI 0.970–1.105)	1.952*** (95% CI 1.538–2.478)	0.639 (95% CI 0.457–0.894)
Urban teaching	0.960 (95% CI 0.928–0.993)	1.155*** (95% CI 1.086–1.228)	3.325*** (95% CI 2.639–4.189)	0.866 (95% CI 0.638–1.176)
Bedsize of hospital				
Medium		1.020 (95% CI 0.964–1.079)	1.442*** (95% CI 1.252–1.660)	
Large		1.059* (95% CI 1.004–1.116)	1.858*** (95% CI 1.626–2.123)	
APR-DRG classification				
Severity of illness				
Moderate	0.892*** (95% CI 0.872–0.913)	0.985 (95% CI 0.936–1.037)	1.278** (95% CI 1.086–1.503)	2.419E + 6 (95% CI NA)
Major	0.750*** (95% CI 0.729–0.772)	1.454*** (95% CI 1.376–1.536)	2.036*** (95% CI 1.729–2.397)	4.126E6 (95% CI NA)
Extreme	0.598*** (95% CI 0.566–0.633)	2.805*** (95% CI 2.620–3.003)	2.897*** (95% CI 2.424–3.462)	5.811E6 (95% CI NA)
Risk of mortality				
Moderate	0.954* (95% CI 0.932–0.976)	1.125*** (95% CI 1.089–1.162)	0.894** (95% CI 0.838–0.953)	5.551*** (95% CI 2.179–14.143)
Major	0.885*** (95% CI 0.850–0.922)	1.379*** (95% CI 1.313–1.449)	0.810*** (95% CI 0.744–0.880)	29.031*** (95% CI 11.497–73.308)
Race				
Black		1.147*** (95% CI 1.097-1.200)	1.108* (95% CI 1.036–1.185)	
Hispanic		1.095*** (95% CI 1.051–1.142)	0.982 (95% CI 0.917–1.051)	
Asian or Pacific Islander		1.084** (95% CI 0.994–1.184)	0.888 (95% CI 0.752–1.049)	
Native American		1.068 (95% CI 0.942–1.211)	1.131 (95% CI 0.903–1.418)	
Other		1.157*** (95% CI 1.069–1.252)	0.895 (95% CI 0.790–1.014)	
Change per year	1.000 (95% CI 0.998–1.001)	0.984*** (95% CI 0.981–0.987)	1.019*** (95% CI 1.014–1.024)	0.953*** (95% CI 0.943–0.962)
Constant	2.020 (95% CI 0.057–71.985)	4.178E+14*** (95% CI 1.326E+12–1.317E+17)	9.092E−19*** (95% CI 4.325E−23–1.912E−14)	2.119E+33 (95% CI 3.391E+24–1.324E+42)
Log likelihood		− 85,047.742		
Theta		2.583*** (0.0276)		
AIC	50,142	170,173	28,270	3709.3

*p < 0.05; **p < 0.01; ***p < 0.001

### Older adults

Older adults received operative care in 3.3% of admissions. Older adults treated operatively were younger, on average, (70.4 vs. 79.6 years, *p* < 0.001) and more commonly male (57.7% vs. 19.6%, *p* < 0.001), less likely to have 12 of the 17 comorbid medical conditions evaluated (absolute percent difference range = 0.5% to 8.5%), but significantly more likely to have mild liver disease and diabetes with complication (absolute percent difference range = 0.4% to 0.5%). Older adults treated nonoperatively were more likely to have Medicare insurance (82.9% vs. 48.0%, *p* < 0.001). Older adults treated with operative management were more likely to receive care at larger hospitals (77.8% vs. 57.4%, *p* < 0.001). Operative care was more likely provided at urban teaching hospitals (87.8% vs. 55.3%, *p* < 0.001). Geographically, operative treatment was more commonly administered in the Southern United States (45.1% vs. 40.2% *p* < 0.001). Per the APR-DRG classification, older adults treated operatively were more frequently classified as having an extreme severity of illness (32.6% vs. 9.6%, *p* < 0.001) and major or extreme risk of mortality (45.0% vs. 30.0%, *p* < 0.001; Table 1).

The in-hospital mortality rate among older adults was 3.5%. The discharge to home rate was 24.1%. The median length of stay was 4 days (IQR = 4 days). Older adults who received operative care stayed in the hospital longer (median 9 vs. 4 days, *p* < 0.001), were less frequently discharged home (18.5% vs. 24.3%, *p* < 0.001), and experienced greater mortality (3.8% vs. 3.5%, *p* < 0.001; Table 1).

The portion of older adults treated operatively increased 0.17% per year (95% CI 0.10–0.24, *p* < 0.001). The home discharge rate decreased − 0.32% per year (95% CI − 0.58 to − 0.06, *p* = 0.02). Length of stay decreased by 0.09% per year (95% CI − 0.12 to − 0.06 days, *p* < 0.001). In-hospital mortality remained stable.

Multivariable analysis demonstrated that the adjusted annual rate of operative care increased by 4.7% per year (95% CI1.035–1.060, *p* < 0.001) among older adults. The adjusted length of stay decreased by 2.0% per year (95% CI 0.977–0.982, *p* < 0.001). The adjusted rate of discharge home decreased by 4.5% (95% CI0.945–0.964, *p* < 0.001) per year. The adjusted in-hospital mortality rate decreased by 0.6% (95% CI 0.991–0.998, *p* = 0.018) per year (Table 3).

**Table 3:** Comparison of multivariable poisson regression models assessing patients over the age of 60 years

Patients aged ≥ 60 years	Dependent variable			
	Home discharge (1)	Length of stay (2)	Operative care (3)	Mortality (4)
Home discharge		1.058*** (95% CI 1.032–1.085)	0.534*** (95% CI 0.470–0.606)	3.686E-08 (95% CI 3.393E-08-4.004E-08)
Length of stay	1.005*** (95% CI 1.003–1.006)		1.010*** (95% CI 1.007–1.014)	0.945*** (95% CI 0.933–0.956)
Operative care	0.582*** (95% CI 0.525–0.646)	1.316*** (95% CI 1.256–1.379)		0.555*** (95% CI 0.431–0.714)
Mortality			0.397*** (95% CI 0.298–0.530)	
Demographics				
Age	0.973*** (95% CI 0.970–0.975)	0.990*** (95% CI 0.987–0.992)	0.955*** (95% CI 0.947–0.964)	1.012*** (95% CI 1.007–1.017)
Female	0.903*** (95% CI 0.869–0.938)	0.907*** (95% CI 0.886–0.929)	0.402*** (95% CI 0.361–0.449)	0.703*** (95% CI 0.644–0.768)
Comorbidities				
CCI Score	0.964** (95% CI 0.943–0.985)	1.022** (95% CI 1.002–1.043)	0.894** (95% CI 0.838–0.954)	
Metastatic solid tumor	1.398*** (95% CI 1.195–1.636)	0.859** (95% CI 0.777–0.950)	0.362 (95% CI 0.110–1.194)	1.290 (95% CI 1.026–1.623)
Liver disease				
Mild		0.936* (95% CI 0.866–1.010)		1.241 (95% CI 1.049–1.470)
Moderate/severe	1.239* (95% CI 1.055–1.454)	0.924 (95% CI 0.809–1.055)		
Malignancy	1.436*** (95% CI 1.302–1.585)	0.955 (95% CI 0.892–1.021)	0.687 (95% CI 0.435–1.084)	
Renal	1.133** (95% CI 1.057–1.214)	0.873*** (95% CI 0.829–0.919)	0.777 (95% CI 0.606–0.996)	0.767*** (95% CI 0.678–0.868)
Hemiplegia/Paraplegia		1.165*** (95% CI 1.006–1.349)	0.648 (95% CI 0.390–1.077)	
Diabetes				
With complication	1.156** (95% CI 1.072–1.247)	1.096*** (95% CI 1.047–1.146)		
Without complication		0.896*** (95% CI 0.864–0.930)	1.253* (95% CI 1.044–1.503)	
PUD	1.337** (95% CI 1.137–1.573)	1.106** (95% CI 0.988–1.237)	0.537 (95% CI 0.239–1.207)	
Rheumatic	1.097* (95% CI 1.016–1.184)	0.959 (95% CI 0.890–1.033)		
COPD	1.082** (95% CI 1.033–1.134)	0.909*** (95% CI 0.883–0.935)	0.829* (95% CI 0.705–0.976)	
Dementia		0.974* (95% CI 0.939–1.011)	0.655** (95% CI 0.501–0.856)	0.847 (95% CI 0.715–1.003)
CVD		0.956* (95% CI 0.914-1.000)		
PVD	1.083* (95% CI 1.017–1.154)	0.959* (95% CI 0.926–0.992)		
CHF	1.167*** (95% CI 1.099–1.240)	0.963 (95% CI 0.927–1.000)	0.736* (95% CI 0.587–0.921)	
MI		0.906*** (95% CI 0.868–0.945)		
Expected primary payer				
Medicaid	1.304*** (95% CI 1.194–1.423)	1.353*** (95% CI 1.245–1.470)	1.149 (95% CI 0.900-1.467)	1.651** (95% CI 1.325–2.057)
Private insurance	1.127*** (95% CI 1.077–1.178)	1.141*** (95% CI 1.105–1.179)	1.553*** (95% CI 1.370–1.760)	1.388*** (95% CI 1.249–1.542)
Self-pay	1.662*** (95% CI 1.519–1.818)	1.329*** (95% CI 1.105–1.599)	1.262 (95% CI 0.917–1.738)	2.144*** (95% CI 1.760–2.611)
No charge	1.503 (95% CI 1.205–1.874)	2.453*** (95% CI 1.526–3.944)	0.795 (95% CI 0.277–2.285)	5.216 (95% CI 1.216–22.382)
Other	1.226*** (95% CI 1.125–1.337)	1.160*** (95% CI 1.089–1.237)	1.487*** (95% CI 1.219–1.815)	1.817*** (95% CI 1.502–2.198)
Region of hospital				
Midwest	1.140*** (95% CI 1.077–1.207)	0.905*** (95% CI 0.881–0.930)	1.077 (95% CI 0.899–1.291)	0.811* (95% CI 0.711–0.924)
South	1.296*** (95% CI 1.234–1.360)	0.978 (95% CI 0.955–1.002)	1.498*** (95% CI 1.290–1.740)	0.873 (95% CI 0.782–0.975)
West	1.352*** (95% CI 1.280–1.427)	0.880*** (95% CI 0.851–0.909)	1.640*** (95% CI 1.397–1.1.924)	0.837* (95% CI 0.738–0.950)
Location/teaching status of hospital				
Urban nonteaching	0.875*** (95% CI 0.830–0.923)	0.917*** (95% CI 0.879–0.956)	1.438 (95% CI 0.970–2.133)	0.796* (95% CI 0.655–0.969)
Urban teaching	0.894*** (95% CI 0.850–0.941)	1.001 (95% CI 0.960–1.043)	4.506*** (95% CI 3.116–6.515)	1.015 (95% CI 0.847–1.217)
Bedsize of hospital				
Medium	0.944 (95% CI 0.898–0.993)	1.016 (95% CI 0.985–1.048)	1.686*** (95% CI 1.303–2.180)	1.058 (95% CI 0.892–1.254)
Large	0.942* (95% CI 0.900–0.986)	1.063*** (95% CI 1.036–1.091)	2.756*** (95% CI 2.166–3.505)	1.162 (95% CI 0.995–1.357)
APR-DRG classification				
Severity of illness				
Moderate	0.798*** (95% CI 0.757–0.843)	0.879*** (95% CI 0.844–0.916)	1.331 (95% CI 0.957–1.850)	0.235*** (95% CI 0.117–0.471)
Major	0.574*** (95% CI 0.538–0.612)	1.055*** (95% CI 1.009–1.103)	2.004*** (95% CI 1.430–2.809)	0.728 (95% CI 0.371–1.429)
Extreme	0.404*** (95% CI 0.357–0.456)	2.015*** (95% CI 1.890–2.148)	3.529*** (95% CI 2.410–5.167)	1.648 (95% CI 0.821–3.309)
Risk of mortality				
Moderate	0.810*** (95% CI 0.776–0.846)	1.240*** (95% CI 1.207–1.273)	0.700*** (95% CI 0.604–0.811)	2.486** (95% CI 1.356–4.556)
Major	0.821*** (95% CI 0.769–0.877)	1.480*** (95% CI 1.426–1.536)	0.750** (95% CI 0.622–0.905)	12.120*** (95% CI 6.521–22.527)
Extreme	0.736*** (95% CI 0.644–0.841)	1.739*** (95% CI 1.628–1.856)	0.759* (95% CI 0.600-0.959)	53.153*** (95% CI 28.285–99.883)
Race				
Black	1.065 (95% CI 0.974–1.164)	1.128*** (95% CI 1.069–1.190)		
Hispanic	1.272*** (95% CI 1.198–1.351)	1.127*** (95% CI 1.072–1.185)		
Asian or Pacific Islander	1.137 (95% CI 1.013–1.278)	1.088*** (95% CI 1.008–1.174)		
Native American	1.099 (95% CI 0.868–1.392)	1.056 (95% CI 0.936–1.191)		
Other	1.160* (95% CI 1.045–1.287)	1.033 (95% CI 0.967–1.104)		
Change per year	0.994* (95% CI 0.991–0.998)	0.980*** (95% CI 0.977–0.982)	1.047*** (95% CI 1.035–1.060)	0.955*** (95% CI 0.945–0.964)
Constant	3.081E+5** (95% CI 131.258–7.231E+8)	5.683E+18*** (95% CI 3.318E+16–9.735E+20)	5.592E−42*** (95% CI 8.248E−53–3.792E−31)	1.062E+38*** (95% CI 1.695E+29–6.652E+46)
Log likelihood				
Theta		***		
AIC	48,008	223,751	9.786.1	8086.2

## Discussion

In this retrospective analysis of US hospital discharge data, operative care of LC1 pelvis fractures increased from 2003 to 2018 by a relative 2% per year among younger adults and 5% per year among older adults after adjusting for factors potentially associated with surgery. Nonoperative care remained, by far, the most common method of treatment of LC1 pelvis fractures over this period. The gradual rise in operative care occurred concurrently with decreases in hospital length of stay and in-hospital mortality in both age groups. Among older adults, however, the increases in operative care occurred concurrently with decreases in home discharge.

The operative or nonoperative management of an LC1 pelvis fracture remains a controversial choice, one influenced by patient factors, injury pattern [15, 16], perceived fracture stability [12–14, 17, 18], historical bias and surgeon preference [19]. Beckmann and colleagues presented a series of LC1 pelvis fractures to 111 Orthopaedic Trauma Association (OTA) surgeons and observed no agreement on operative or nonoperative care in two-thirds of the cases [15]. Vallier and colleagues prospectively observed that LC1 pelvis fractures of similar morphology were treated operatively or nonoperatively by different surgeons [19]. Nonoperative care consistently has been shown to provide satisfactory rates of fracture union [8, 20, 21]. In a systematic review, Varma et al. noted no significant differences in patient reported outcomes, length of stay, or complication rates among younger patients [22]. Conversely, recent observational studies suggest that operative care of LC1 pelvis fractures may be associated with better outcomes in length of hospital stay, mobilization, and pain control, particularly among older adults [12, 21]. Slobogean and colleagues prospectively observed minor benefits with regard to pain with operative care among younger patients [9].

Operative care has been described as an intervention that may improve the probability of home discharge [10, 22–24]. Discharge home after pelvis fracture is highly valued by patients [14] and has been demonstrated to have fewer complications, fewer readmissions, and lower costs following hip fractures [25]. However, 15 years of hospital discharge data refute this assumption when adjusting for factors associated with surgery and with discharge destination. Operative care was not associated with discharge destination for younger adults and was associated with non-home discharge among older adults.

Operative care may have a causal effect on length of hospital stay, but may also reflect confounding by indication for injury severity and/or treatment algorithm. Patients of all ages who underwent operative management had significantly longer hospital stays than those who underwent nonoperative care. Since APR-DRG classification reflects acute inpatient illness severity and not simply comorbidity status, patients undergoing operative care possessed higher APR-DRG scores but lower comorbidity rates, suggesting higher energy mechanisms and more severe injuries may have contributed to longer inpatient courses. Similarly, the timing of operative care likely has a causal effect on length of stay: nonoperative observation until offering surgery when the patient fails to mobilize after a certain number of days may prolong hospital stay compared with early internal fixation. Conversely, early definitive internal fixation may or may not shorten length of stay compared with nonoperative care. Booth et al., in a systematic review of LC1 fragility fractures, conversely found operative fixation was significantly associated with longer length of stay [23]. However, Varma et al. in a systematic review of LC1 injuries with complete sacral fractures found no difference in length of stay by treatment among younger adults [22]. Tosounidis et al. reported patients who underwent operative fixation after LC1 fracture with complete sacral fracture had significantly shorter inpatient hospital stays compared to those with incomplete sacral fractures who underwent nonoperative management [12]. It is not possible to ascertain from the NIS data if the severity of fracture patterns, concomitant injuries, or timing of surgery relative to date of admission significantly changed over the observation period.

The rates of mortality observed after LC1 pelvis fracture in this cohort were lower than previously reported and may support operative management. In-hospital mortality of 3–4% and a 5% relative decrease in mortality per year markedly differed from the 5%-9% mortality rate reported after LC1 fracture reported by Burgess and colleagues [4]. Höch and colleagues also observed decreased odds of mortality with operative management of lateral compression pelvis fractures in older adults [3]. Furthermore, the macro trends observed in this study parallel geriatric hip fracture literature demonstrating decreased mortality and length of stay from 2010 to 2019, potentially suggesting these changes may be secondary to system-level improvement in healthcare delivery [26].

There are limitations to this study inherent to a retrospective database study including sampling, classification, and observation bias limited to the index admission. Without radiographic verification of injury pattern, the coding strategy to capture LC1 pelvic fractures is a best-approximation of diagnoses codes excluding concurrent lumbar vertebrae injuries, ilium fractures, and open fractures. The diagnosis-based selection strategy introduces classification bias that systematically may alter the accuracy of point estimates, but may marginally affect the precision of temporal trends in relative observations. Data from 2015 and after may skew Charlson Comorbidity Index and outcome estimate errors due to the transition from ICD-9 to ICD-10 coding. However, this study is strengthened by the use of a national database, thereby increasing generalizability.

In conclusion, US hospital discharges observed in the NIS database indicate that operative care of LC1 pelvis fractures increased from 2003 to 2018. A rise in operative care was associated with decreases in length of stay and mortality in older and younger adults, but fewer home discharges for older adults.

## Supplementary Information

Below is the link to the electronic supplementary material.

Supplementary Material 1.

Supplementary Material 2.

## Data Availability

The data generated in this study is available upon reasonable request.
