# Supplementary material for: Operative care rates of lateral compression type 1 pelvic fractures increased from 2003 to 2018
Source: Eur J Orthop Surg Traumatol. 2026 Aug 1;36(1):309. doi: 10.1007/s00590-026-04900-0 (PMC13428703; doi:10.1007/s00590-026-04900-0)
Supplement: Supplementary file 1 — Supplementary Material 1. [file 590_2026_4900_MOESM1_ESM.docx]

Supplementary Table 1. International Classification of Diseases Ninth Edition Clinical Modification (ICD-9) and Tenth Edition (ICD-10) Clinical Modification codes used for diagnosis of closed LC1 pelvic fractures.

|  | ICD-9 and ICD-10 diagnosis codes |
| --- | --- |
| Closed LC1 pelvic fractures | ICD-10-D-S3210XA, ICD-10-D-S3210XD, ICD-10-D-S3210XG, ICD-10-D-S3210XK, ICD-10-D-S3210XS, ICD-10-D-S32110A, ICD-10-D-S32110D, ICD-10-D-S32110G, ICD-10-D-S32110K, ICD-10-D-S32110S, ICD-10-D-S32111A, ICD-10-D-S32111D, ICD-10-D-S32111G, ICD-10-D-S32111K, ICD-10-D-S32111S, ICD-10-D-S32112A, ICD-10-D-S32112D, ICD-10-D-S32112G, ICD-10-D-S32112K, ICD-10-D-S32112S, ICD-10-D-S32119A, ICD-10-D-S32119D, ICD-10-D-S32119G, ICD-10-D-S32119K, ICD-10-D-S32119S, ICD-10-D-S32120A, ICD-10-D-S32120D, ICD-10-D-S32120G, ICD-10-D-S32120K, ICD-10-D-S32120S, ICD-10-D-S32121A, ICD-10-D-S32121D, ICD-10-D-S32121G, ICD-10-D-S32121K, ICD-10-D-S32121S, ICD-10-D-S32122A, ICD-10-D-S32122D, ICD-10-D-S32122G, ICD-10-D-S32122K, ICD-10-D-S32122S, ICD-10-D-S32129A, ICD-10-D-S32129D, ICD-10-D-S32129G, ICD-10-D-S32129K, ICD-10-D-S32129S, ICD-10-D-S32130A, ICD-10-D-S32130D, ICD-10-D-S32130G, ICD-10-D-S32130K, ICD-10-D-S32130S, ICD-10-D-S32131A, ICD-10-D-S32131D, ICD-10-D-S32131G, ICD-10-D-S32131K, ICD-10-D-S32131S, ICD-10-D-S32132A, ICD-10-D-S32132D, ICD-10-D-S32132G, ICD-10-D-S32132K, ICD-10-D-S32132S, ICD-10-D-S32139A, ICD-10-D-S32139D, ICD-10-D-S32139G, ICD-10-D-S32139K, ICD-10-D-S32139S, ICD-10-D-S3214XA, ICD-10-D-S3214XD, ICD-10-D-S3214XG, ICD-10-D-S3214XK, ICD-10-D-S3214XS, ICD-10-D-S3215XA, ICD-10-D-S3215XD, ICD-10-D-S3215XG, ICD-10-D-S3215XK, ICD-10-D-S3215XS, ICD-10-D-S3216XA, ICD-10-D-S3216XD, ICD-10-D-S3216XG, ICD-10-D-S3216XK, ICD-10-D-S3216XS, ICD-10-D-S3217XA, ICD-10-D-S3217XD, ICD-10-D-S3217XG, ICD-10-D-S3217XK, ICD-10-D-S3217XS, ICD-10-D-S3219XA, ICD-10-D-S3219XD, ICD-10-D-S3219XG, ICD-10-D-S3219XK, ICD-10-D-S3219XS, ICD-10-D-S32810A, ICD-10-D-S32810D, ICD-10-D-S32810G, ICD-10-D-S32810K, ICD-10-D-S32810S, ICD-10-D-S32811A, ICD-10-D-S32811D, ICD-10-D-S32811G, ICD-10-D-S32811K, ICD-10-D-S32811S, ICD-10-D-S3282XA, ICD-10-D-S3282XD, ICD-10-D-S3282XG, ICD-10-D-S3282XK, ICD-10-D-S3282XS, ICD-9-D-8056, ICD-9-D80843, ICD-9-D80844 |
| Exclusion codes | ICD-10-D-S3210XB, ICD-10-D-S32110B, ICD-10-D-S32111B, ICD-10-D-S32112B, ICD-10-D-S32119B, ICD-10-D-S32120B, ICD-10-D-S32121B, ICD-10-D-S32122B, ICD-10-D-S32129B, ICD-10-D-S32130B, ICD-10-D-S32131B, ICD-10-D-S32132B, ICD-10-D-S32139B, ICD-10-D-S3214XB, ICD-10-D-S3215XB, ICD-10-D-S3216XB, ICD-10-D-S3217XB, ICD-10-D-S3219XB, ICD-10-D-S32810B, ICD-10-D-S32811B, ICD-10-D-S3282XB, ICD-10-D-S320, ICD-10-D-S323, ICD-9-D8054, ICD-9-8055 |
