# Supplementary material for: Operative care rates of lateral compression type 1 pelvic fractures increased from 2003 to 2018
Source: Eur J Orthop Surg Traumatol. 2026 Aug 1;36(1):309. doi: 10.1007/s00590-026-04900-0 (PMC13428703; doi:10.1007/s00590-026-04900-0)
Supplement: Supplementary file 2 — Supplementary Material 2. [file 590_2026_4900_MOESM2_ESM.docx]

Supplementary Table 2. ICD-9 and ICD-10 procedure codes used for surgical treatment of pelvic fractures.

|  | ICD-9-CM and ICD-10-CM procedure codes |
| --- | --- |
| Operative management of pelvic fractures | ICD-10-P-0QH204Z, ICD-10-P-0QH205Z, ICD-10-P-0QH304Z, ICD-10-P-0QH305Z, ICD-10-P-0QQ20ZZ, ICD-10-P-0QQ30ZZ, ICD-10-P-0QS204Z, ICD-10-P-0QS205Z, ICD-10-P-0QS20ZZ, ICD-10-P-0QS304Z, ICD-10-P-0QS305Z, ICD-10-P-0QS30ZZ, ICD-10-P-0QQ2XZZ, ICD-10-P-0QQ3XZZ, ICD-10-P-0QS2XZZ, ICD-10-P-0QS3XZZ, ICD-10-P-0QQ1XZZ, ICD-10-P-0QS1XZZ, ICD-10-P-0QH234Z, ICD-10-P-0QH235Z, ICD-10-P-0QH334Z, ICD-10-P-0QH335Z, ICD-10-P-0QQ23ZZ, ICD-10-P-0QQ33ZZ, ICD-10-P-0QS234Z, ICD-10-P-0QS235Z, ICD-10-P-0QS23ZZ, ICD-10-P-0QS334Z, ICD-10-P-0QS335Z, ICD-10-P-0QS33ZZ, ICD-10-P-0QH134Z, ICD-10-P-0QH135Z, ICD-10-P-0QQ13ZZ, ICD-10-P-0QS134Z, ICD-10-P-0QS13ZZ, ICD-10-P-0QH244Z, ICD-10-P-0QH245Z, ICD-10-P-0QH344Z, ICD-10-P-0QH345Z, ICD-10-P-0QQ24ZZ, ICD-10-P-0QQ34ZZ, ICD-10-P-0QS244Z, ICD-10-P-0QS245Z, ICD-10-P-0QS24ZZ, ICD-10-P-0QS344Z, ICD-10-P-0QS345Z, ICD-10-P-0QS34ZZ, ICD-10-P-0QH144Z, ICD-10-P-0QH145Z, ICD-10-P-0QQ14ZZ, ICD-10-P-0QS144Z, ICD-9-P-7810, ICD-9-P-7819, ICD-9-P-791, ICD-9-P-7919, ICD-9-P-792, ICD-9-P-7929, ICD-9-P-793, ICD-9-P-7939, ICD-9-P-785, ICD-9-P-7859 |
